# Supplementary material for: Determinants of regulatory compliance in health and social care services: A systematic review using the Consolidated Framework for Implementation Research
Source: PLoS One. 2023 Apr 13;18(4):e0278007. doi: 10.1371/journal.pone.0278007 (PMC10101495; doi:10.1371/journal.pone.0278007)
Supplement: Attachment — Submitted filename: Review PONE-D-22-30687.docx [file pone.0278007.s007.docx]

The study presents the results of original research.
2. Results reported have not been published elsewhere.
3. Experiments, statistics, and other analyses are performed to a high technical standard and are described in sufficient detail.
4. Conclusions are presented in an appropriate fashion and are supported by the data.
5. The article is presented in an intelligible fashion and is written in standard English.
6. The research meets all applicable standards for the ethics of experimentation and research integrity.
7. The article adheres to appropriate reporting guidelines and community standards for data availability.

**Review**

Thank you for submitting this systematic review which adds to the literature on factors affecting compliance. The article which is interesting, detailed and well written and has a published protocol.

I have included some minor suggestions below

Only 20 of the existing 39 CIFR constructs were used. There is excessive used of an ‘other’ category Some of the determinants mentioned need to be moved to the relevant constructs.

**Abstract**

The writing needs to be consistent. A mixture of third person and first person styles are used e.g.

Results: The search yielded.. We found……

**Introduction**

**Methods**

Line 101. Needs to include a reference to regulatory compliance rather than just ‘compliance’

Analysis

Line 141. Explain what is meant by ‘different’ for nominal variables.

**Results:**

It would be helpful to indicate the number of constructs within each of the 5 headings (It is clear for inner setting) so that it is evident how may constructs out of the total are used in each domain.

Table 1.

Suggest to regroup European countries together in alphabetical order under ‘Country’ as n=1 in all cases.

Complexity Lines 265-6. Brief detail on the type of regulatory measure referred to here would help to explain why it would need its own QA meeting.

Line 285-286. The external influences referred to here are not just those that related to the needs and recourses of service users.

External policy and incentives includes funding. The point under ‘Other’ which relates to funding, Lines 351-353 should be moved here.

Lines 354-358 are External influences and should be moved to that section

**Discussion**

Some discussion on the mixed quality of the included studies is needed.

Reference to the updated version of CIFR Damschroder et al. Implementation Science (2022) 17:75https://doi.org/10.1186/s13012-022-01245-0 needs to be included.
